# CD46 knock-out using CRISPR/Cas9 editing of hTERT immortalized human cells modulates complement activation

**DOI:** 10.1371/journal.pone.0214514

**Published:** 2019-04-08

**Authors:** Matthias Wieser, Teresa Francisci, Daniel Lackner, Tilmann Buerckstuemmer, Kamilla Wasner, Wolf Eilenberg, Anton Stift, Markus Wahrmann, Georg A. Böhmig, Johannes Grillari, Regina Grillari-Voglauer

**Affiliations:** 1 Evercyte GmbH, Vienna, Austria; 2 Horizon Genomics GmbH, Vienna, Austria; 3 Department of Medicine III, Division of Nephrology and Dialysis, Medical University of Vienna, Vienna, Austria; 4 Department of Surgery, Medical University of Vienna, Vienna, Austria; 5 Department of Biotechnology, BOKU Vienna, Vienna, Austria; 6 Austrian Cluster for Tissue Regeneration, Vienna, Austria; 7 Christian Doppler Laboratory for Biotechnology of Skin Aging, Vienna, Austria; University of Newcastle, UNITED KINGDOM

## Abstract

The kidney is especially sensitive to diseases associated with overactivation of the complement system. While most of these diseases affect kidney glomeruli and the microvasculature, there is also evidence for tubulointerstitial deposition of complement factors. Complement inactivating factors on cell membranes comprise CD55, CD59 and CD46, which is also termed membrane cofactor protein (MCP). CD46 has been described as localized to glomeruli, but especially also to proximal tubular epithelial cells (RPTECs). However, human cell culture models to assess CD46 function on RPTECs are still missing. Therefore, we here performed gene editing of RPTEC/TERT1 cells generating a monoclonal CD46^-/-^ cell line that did not show changes of the primary cell like characteristics. In addition, factor I and CD46-mediated cleavage of C4b into soluble C4c and membrane deposited C4d was clearly reduced in the knock-out cell line as compared to the maternal cells. Thus, human CD46^-/-^ proximal tubular epithelial cells will be of interest to dissect the roles of the epithelium and the kidney in various complement activation mediated tubulointerstitial pathologies or in studying CD46 mediated uropathogenic internalization of bacteria. In addition, this gives proof-of-principle, that telomerized cells can be used in the generation of knock-out, knock-in or any kind of reporter cell lines without losing the primary cell characteristics of the maternal cells.

## 2. Introduction

Several kidney diseases are associated with the complement system. Thereby, a majority of conditions shows complement deposition in endothelium and glomeruli, but there is also emerging evidence of tubulointerstitial complement factor depositions. For instance, non-selective proteinuria causes a massive burden of C3 deposition to proximal tubuli cells posing a considerable challenge to locally expressed complement regulatory proteins [1]. In addition, it is now clear, that the kidney itself is an important source of complement factors, with estimates of about 10% of circulating C3 being derived from kidney [2], while the majority of the complement factors circulating in blood are derived from liver. Indeed, all complement factors and complement regulating proteins have been found to be constitutively and/or inducibly expressed in different kidney cells in vitro and in vivo [3]. Thereby, renal proximal tubular epithelial cells (RPTECs) seem to be a major source of renal complement production. This, however, requires protection of RPTECs themselves from autologous activation of complement, highlighting an important role for complement inactivating factors on these cells.

One complement regulating factor that has gained interest in this regard as well as in the context of kidney transplantation [4], atypical hemolytic uremia (aHUS) [5], or uptake of uropathogenic bacteria [6], is CD46, also termed membrane cofactor protein (MCP). In other tissues than kidney it is hitchhiked as a virus entry point for measles, HHV6, herpes simplex [7], but also for bacterial entry by Neisseria meningitis [8]. In kidney, it is differentially expressed in diseased versus healthy kidney [9].

CD46 / MCP is a co-factor of factor I responsible for cleaving C4b and C3b - which were unintendedly deposited onto self membrane surfaces—into less harmful split products. While ubiquitously expressed in human tissues, in the kidney, CD46 is present in the glomerular apparatus, collecting duct and tubuli [10], especially at the basolateral side of proximal tubular cells as well as in interstitial cells. CD46 importance in RPTECs is underlined by the notion that the two other factors protecting tissues against complement induced damage, CD59 and CD55 are expressed at lower levels where CD46 is high [11]^,^[12]. In addition, low expression of CD46 in proximal tubuli is associated with the inflammatory disease antibody mediated vasculitis [13] and proximal tubular epithelial cells have been postulated to be pro-inflammatory cells [2]. However, elucidating the role of CD46 in these cells is hampered as in mouse models, the mouse homologous CD46 is restricted to testis tissue and the retina [14], while in kidney and other tissues its function seems to be substituted by Crry gene. Nevertheless, transgenically human CD46 expressed in mice shows intense staining in renal tubuli [15]. Experimental models, especially gene knock-out models, to study the role of human CD46 in RPTECs are therefore limited. In addition, the use of primary or normal human cells for generating knock-out cells is severely hampered by the limited replicative life span that is due to progressive telomere shortening [16]. As a means to counteract entry into senescence, expression of the catalytic subunit of human telomerase (hTERT) has been successfully used and is by now well acknowledged to only minimally change the characteristics of hTERT immortalized cells [17]. Among the cells that are amenable to hTERT immortalization are fibroblasts [18], endothelial cells [19,20], adipose and bone marrow derived MSCs [21,22] or renal tubular epithelial cells (RPTEC/TERT1) [23].

We here selected the highly differentiated RPTEC/TERT1 cells [24,25] in order to perform CD46 knock-out using the CrispR/Cas9 system. Therefore, we optimized transfection, subcloning and growth conditions in order to efficiently generate a CD46^-/-^ RPTEC/TERT1 cell line that maintains its primary cell characteristics but also shows reduced ability to deactivate C4b.

We expect that the combination of hTERT immortalization and gene editing will be of broad interest, whenever gene knock-out is studied in normal cell function, whenever reporter constructs need to be stably integrated into specific loci like e.g. the human analogue to the mouse Rosa26 locus [26], or whenever specific knock-ins, e.g. of mutant single nucleotide polymorphisms (SNPs), need to be generated in order to functionally understand results from e.g. genome wide association studies (GWAS) [27].

## 3. Results

### 3.1. Knock-out of CD46

**Fig 1:**
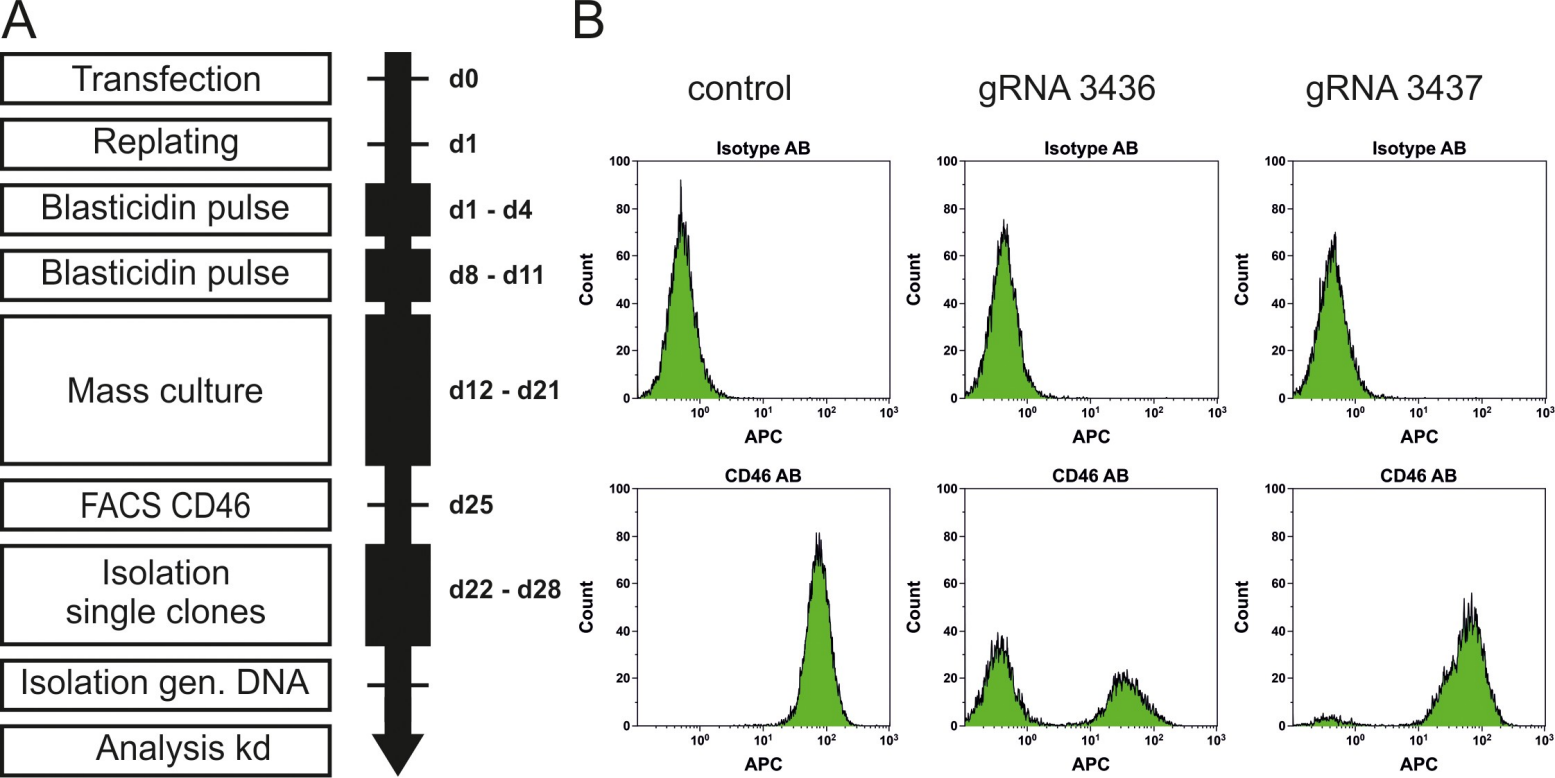
Generation of CD46-/- RPTEC/TERT1 mass culture. **a** Workflow for generation of RPTEC/TERT1 CD46 knock-out cells. **b** FACS analysis of CD46 knock-out in mass culture for gRNA3436 and gRNA3437.

In order to generate a CD46 knock-out RPTEC/TERT1 cell line, transfection efficiency as well as subcloning efficiencies as well as antibiotic treatment for selection using blasticidin were optimized (scheme of Fig 1A). Since we observed a cloning efficiency too low for cloning by limiting dilution, we decided to use colony picking techniques. We then performed CRISPR/Cas9 transfection of RPTEC/TERT1 cells with 2 different guide RNAs, gRNA3436 and gRNA3437. Selection was performed by two 72 hour pulses with 15 μg/mL blasticidin. 25 days after transfection, mass culture of arising clones was analyzed for CD46 expression by flow cytometry. While cells transfected with Cas9 and GFP-Blast plasmid alone as negative control showed uniform CD46 expression, additional transfected with gRNA3436 or gRNA3437 resulted in a clearly distinct CD46 negative sub-population of 57% of the cells using gRNA3436, while 6% only using gRNA3437 (Fig 1B). Individual clones were isolated by mechanical detachment using pipettes. In total 13 clones were picked for gRNA3436, 14 clones for gRNA3437 and 14 control clones. Individual clones were transferred into 96 well plates and further expanded for sequencing, freezing, and characterization.

### 3.2. Characterization of CD46 subclones

**Fig 2:**
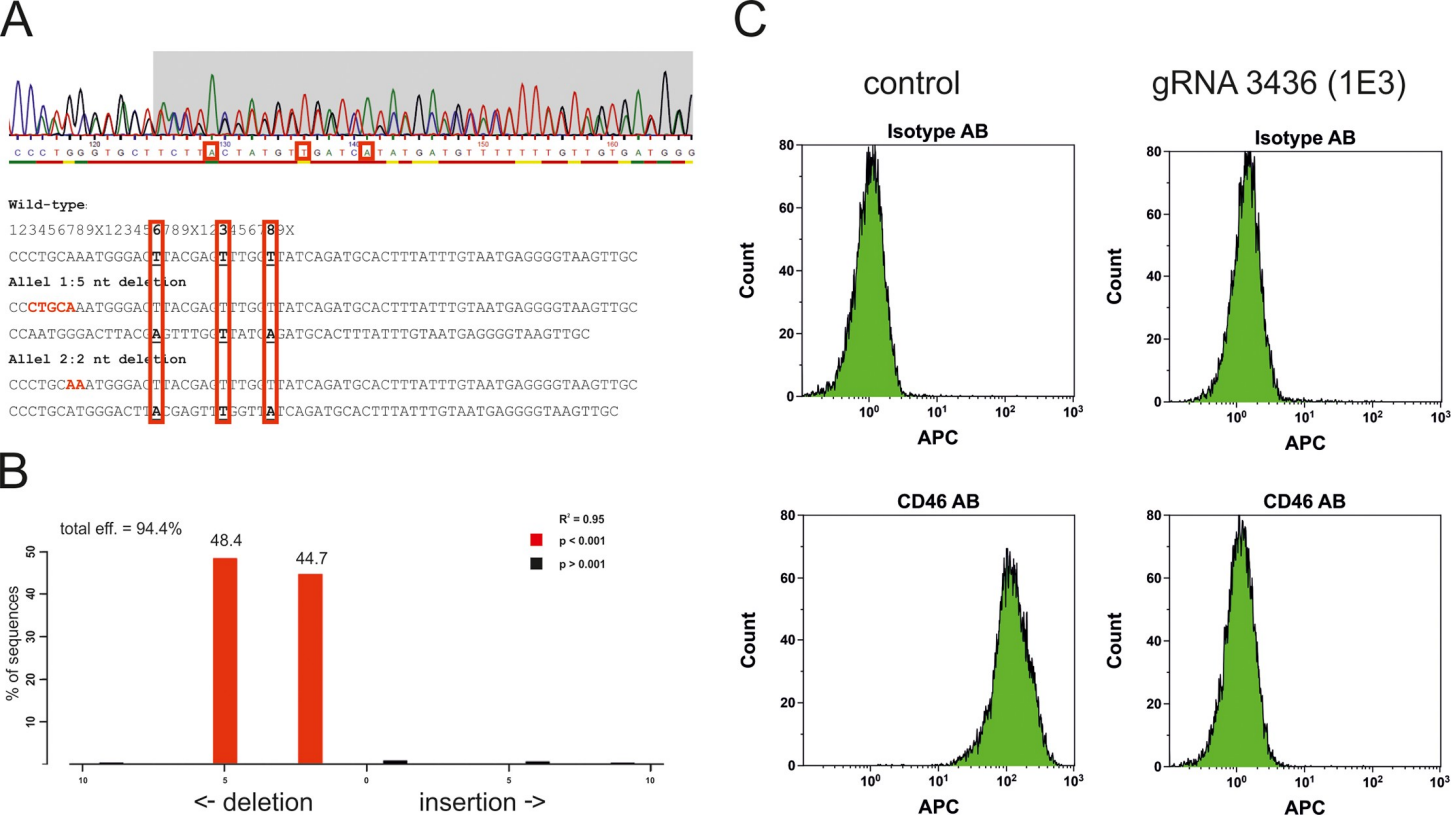
Confirmation of homozygous knock-out of CD46 -/- RPTEC/TERT1 cells. **a** Sanger sequencing results of gene knock-out. Manual alignment. **b** Sanger sequencing results of gene knock-out. Analysis of in-del by TIDE online tool. **c** Confirmation of CD46 knock-out in clone 1E3 on protein level by FACS analysis.

In order to confirm CD46 knock-out, individual clones were expanded to sufficient numbers to isolate genomic DNA. Primers flanking the gRNA binding site were used to amplify this specific part of the CD46 locus. PCR products of 10 clones out of the 41 individual clones isolated in total were analyzed by Sanger sequencing. Overlapping reads where regarded as putative knockouts and were aligned manually in order to test which putative deletions would result in identical base calls at distinct positions (red frames Fig 2A). In a second step hypothesized deletions were disentangled and verified using the TIDE (Tracking of Indels by DEcomposition) online software tool [28]. Of the 10 clones analyzed 7 were transfected with gRNA3437 and of these 7 clones 6 turned out to be wild-type not showing genetic modifications like Indels at the CD46 locus. The remaining clone showed a one nucleotide deletion on one allele whereas the other allele was wild-type (S1B Fig). Of the 3 clones transfected with gRNA3436 analyzed 2 showed homozygous deletions in the CD46 locus whereas 1 sequence was not clear and was not further analyzed. One of the two clones (1E3) with homozygous deletions carried a two nucleotide deletion on the one allele and a five nucleotide deletion on the other (Fig 2A and 2B). The second (1C3) carried a one nucleotide deletion on the one allele and a two nucleotide deletion on the other allele (S1A Fig). Due to this high success rate for gRNA3436, no further clones were analyzed. Finally, we wanted to confirm the sequencing results on protein level. Therefore, clone 1E3 was expanded and analyzed for CD46 expression by cytometry. Control transfected cells that were confirmed to be wild-type by sequencing showed homogenous CD46 expression, whereas in 1E3 RPTEC/TERT1 cells no CD46 positive cells were detected (Fig 2C), thus now termed RPTEC/TERT1 CD46^-/-^.

**Fig 3:**
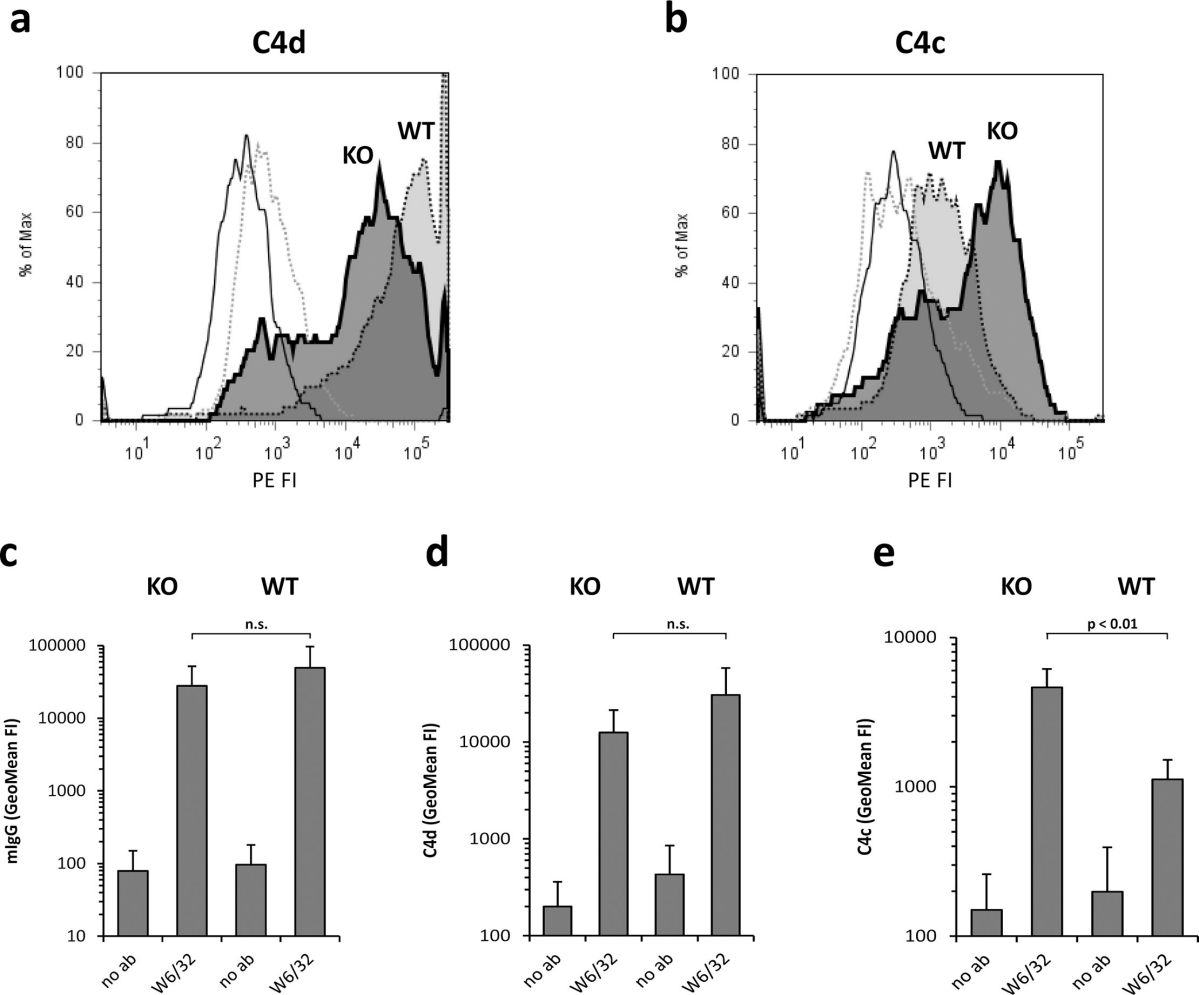
Relative loss of factor I cofactor activity in CD46 -/- RPTEC/TERT1 cells. **a+b** Overlay histograms show C4d **(a)** or C4c **(b)** deposition after classical complement pathway activation on wild-type (WT; control-transfected) and knock-out (KO; 1E3) RPTEC/TERT1 cells. KO: dark-grey tinted area with solid line, WT: light-grey tinted area with dotted line, KO without W6/32 as trigger of classical pathway activation: solid line, WT without W6/32: dotted line. **c** Detection of anti-HLA class I (W6/32) antibody on CD46 -/-(KO) and wild-type (WT) RPTEC/TERT1 cells. **d** Detection of C4d deposition. **e** Detection of C4c deposition. **c-e** Mean values and standard deviations (T-shaped whiskers) of geometric mean of fluorescence intensity of 4 experiments are shown. No ab = without W6/32 in the incubation mixture (only 50% serum) PE phycoerythrin, FI fluorescence intensity, n.s. not significant.

A main functional role of CD46 is the support of complement factor I during digestion of C4b and C3b, a regulatory step that protects own surfaces from establishment of C3 and C5 convertases and thus from further complement activation. In a functional assay we compared the cofactor activity of control transfected maternal cells and 1E3 cells analyzing the degradation of C4b into membrane-bound C4d and released C4c (Fig 3). Both cell types showed remarkable amounts of classical pathway triggering anti-HLA antibody (Fig 3C) and a total sum of complement split products C4b and C4d (measured by anti-C4d; Fig 3A and 3D) deposited on their surfaces. However, there was a relative increase of C4b signal (measured by anti-C4c; Fig 3B and 3E) on RPTEC/TERT1 CD46^-/-^ cells compared to maternal wild-type cells which had only residual anti-C4c signals after one hour of incubation (p = 0.004). In an analogous C3 assay a functional difference between knock-out and wild-type cells could not be demonstrated: mean C3b/d deposition (measured by anti-C3d) on RPTEC/TERT1 CD46^-/-^ cells was 9138 ± 1973 geometric mean of fluorescence intensity (geoMFI) and 10063 ± 4818 on wild-type cells (p = 0.67), and the C3c signal was slightly lower, but without statistical significance, on wild-type than on CD46^-/-^ cells (13420 ± 3300 versus 14627± 5164, p = 0.64).

Overall, these data demonstrate that the generation of homozygous knock-outs of immortalized cells was highly efficient. It is of notice that already analysis of mass culture at a very early time point indicated that gRNA3436 was more efficient than gRNA3437, a result that was confirmed in individual clones, underlining the importance of good design of guide RNA.

**Fig 4:**
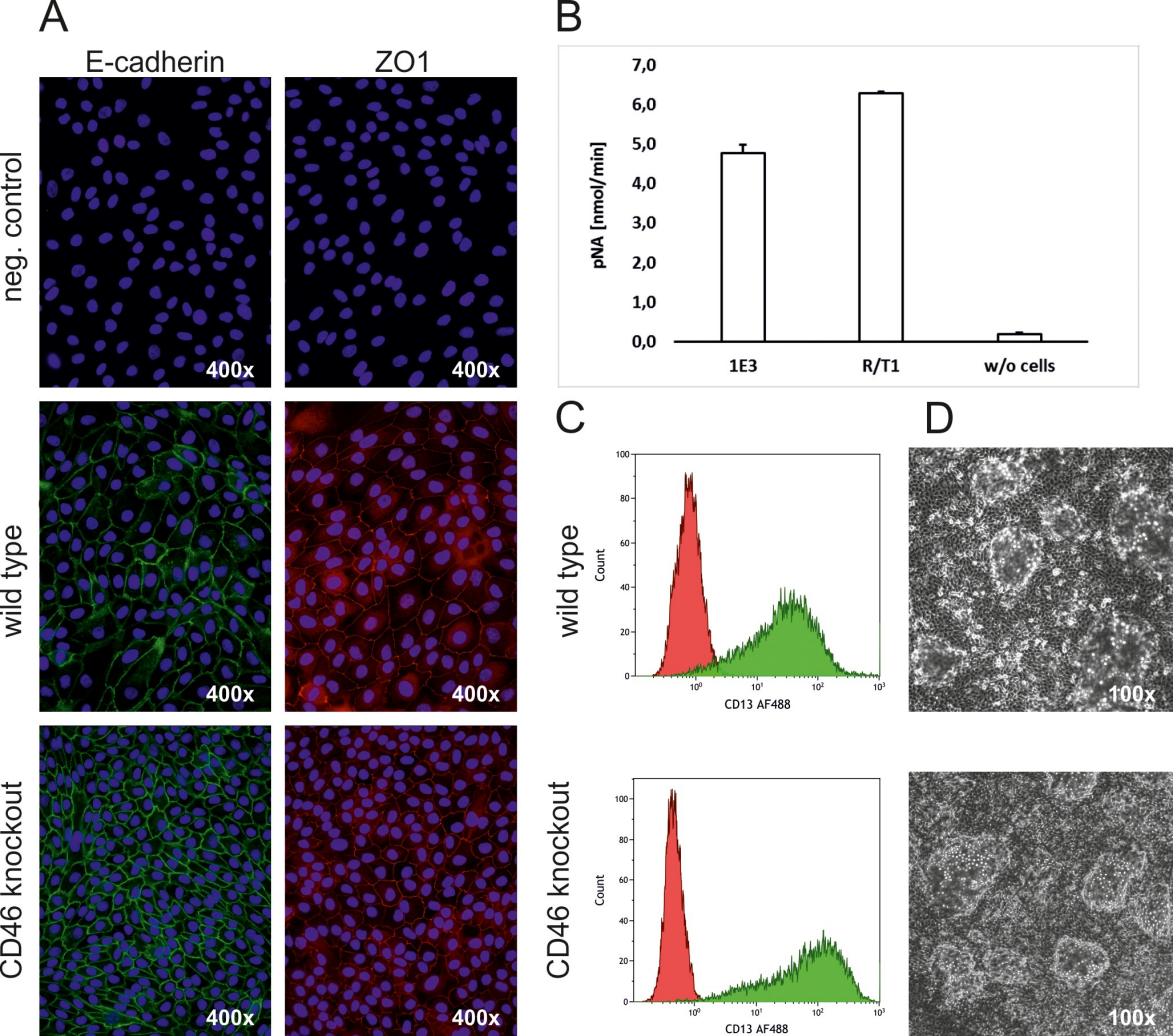
Characterisation of RPTEC/TERT1 CD46 knock-out clone 1E3. **a** Immunofluorescence of E-cadherin and ZO1. **b** Gamma-glutamyltransferase activity. **c** FACS analysis of CD13 (Aminopeptidase N). D: Dome formation.

In order to confirm that cell-type specific characteristics were maintained in RPTEC/TERT1 CD46^-/-^cells, we confirmed their typical cell morphology using light microscopy and their ability to form domes when grown to confluence (Fig 4D). Additionally, the cells still express the renal proximal tubular epithelial surface marker CD13 (Fig 4C), a brush boarder enzyme of the proximal tubuli. Similarly, they exhibited gamma glutamyltransferase (GGT) activity, an enzyme activity characteristic for RPTECs (Fig 4B). Finally, cells showed clear membrane associated E-Cadherin and ZO1 expression (Fig 4A), which, together with dome formation, indicates functional tight junctions, a key characteristic of proximal tubular epithelial cells.

Summarized, these data suggest that the original cellular phenotype was maintained after knocking-out CD46 and the consequently necessary cell expansion of single clones.

## 4. Discussion

Here we report the generation of a CD46 knock-out RPTEC cell line. Gene editing of primary human cells using CRISPR/Cas9 [29], TALENs [30], or even homologous recombination [31] is hugely challenging at best and merely impossible in general for most cell types due to the short replicative life span of primary cells. Depending on the cell type and species, a primary cell can undergo a limited number of population doubling ranging between 2 and 80 PDL before entering replicative senescence due to critically short telomeres. This irreversible cell cycle arrest is exerted via the p53/p21 and/or Rb/p16 pathways resembling a DNA damage checkpoint response [32].

Therefore, only few examples of gene editing of primary cells to generate stable cell strains exist. Among these are human diploid fibroblasts that have been subjected to homologous recombination, and those approaches targeted cell cycle arrest proteins like p21 [33], or retinoblastoma protein [34] for knock-out, resulting in continuously growing cells after successful gene targeting. From these approaches it is clear that gene editing uses up many population doublings during the selection and clonal production phase, so that eventually no or very few PDs remain for using the cells as model systems. Therefore, there is a need for cell models that grow continuously while retaining their parental cell specific characteristics. Indeed, hTERT has been widely used to immortalize cells with minimal or no changes in cellular phenotype and functionality over up to 100 PDs [18,20,22,23]. Alternatively, hTERT overexpression in combination with factors interfering with cell cycle control pathways such as ectopic expression of CDK4 [35–37] or viral oncogenes such as the ones derived from Simian Virus 40 can be used [38–40].

Here we give proof-of-principle that cells immortalized by ectopic expression of hTERT can be edited at their genomic level using the CRISPR/Cas9 system resulting in primary-like continuously growing knock-out cell lines.

Due to the lack of human CD46 renal cell model systems, we selected this gene to be knocked-out on RPTEC cells. It is clear however, that alternative approaches can be applied to generate CD46 defective cell lines, e.g. from patients suffering from CD46 mutation dependent diseases [41–43], or, if the renal phenotype is not of prioritiy to use HAP1 cells as recently used to knock-out CD46 and other genes [44].

The main function of CD46 is to inactivate the complement system and thus to protect host cells from being damaged by the innate immune system [45]. This function is exerted by its supporting of factor I protease activity towards the 2 complement factors C4b and C3b [46]. Accordingly, we could demonstrate a loss of function regarding C4b degradation on CD46^-/-^ cells, however, there was no difference in cleavage of C3b between knock-out and wild-type cells. In this functional assay additional receptors (like CR1) capable of final iC3b cleavage into C3dg and C3c are necessary [47,48][9,49,50]. We speculate that in our *in vitro* assay strong classical complement activation and subsequent excessive C3 amplification loop activation overwhelmed CD46 on wild-type and these remaining complement receptors on CD46^-/-^ cells as well–to such an extent that no significant difference between the two cell lines could be observed.

In addition, CD46 also seems to be a connection point of the innate and adaptive immune system [51], and itself is also regulated by various inflammatory cytokines like IL-1beta, IL-4, and TGF-β, which downregulate CD46 expression in RPTECs and renal tumor cell lines [52]. It has furthermore been identified to play a role in various infectious diseases, as it acts as a receptor and therefore entry point of various human pathogens including measles [7] or some types of uropathogenic E. coli [6]. In kidney tumors, it has been found to protect cancer cells from being attacked by the complement system [53,54]. Finally, porcine kidneys transgenically expressing human CD46 have been considered historically to replace human organ transplants [55]. This is in line with the idea that one of the enzymatic products of CD46, membrane deposited C4d, appears to be protective in ABO-incompatible kidney transplantation [56].

Taken together, we here established CD46 knock-out RPTEC/TERT1 cell line that will be useful in understanding and dissecting the role of RPTECs and the complement in host-pathogen interactions including viral infections or enterobacterial infections including EHEC.

In addition, we have shown that telomerized, primary-like cells are amenable to gene editing. This opens up a wide array of possibilities to generate homozygous knock-out or knock-in cell lines for studying gene function, target and drug identification, or phenotypic drug screening using orthogonal screening strategies for minimizing false positive hits.

## 5. Materials and methods

### 5.1. Cell culture

RPTEC/TERT1 cells were routinely grown on non-coated plastic tissue culture ware in ProxUp serum-free medium (Evercyte GmbH). 72 hours before transfection cells were seeded at a split ratio of 1:2 into 60 mm petri dishes (Greiner BioOne) so that the cells were 95% confluent at time of transfection. Transfection was performed using Lipofectamine 2000 (Thermo Fisher Scientific) with a ratio of lipids to DNA of 1:4.5. The DNA consisted of plasmids coding for Cas9, GFP-Blast and gRNA3436 or gRNA3437 (Horizon Genomics). Selection of transfected cells was achieved by supplementing ProxUp medium with 15 μg/mL blasticidin (InvivoGen).

### 5.2. Gene editing

Gene editing was done using a combination of three plasmids. Individual plasmids coding for Cas9 protein, blasticidin resistance including a GFP cassette to monitor success of transfection and one of two gRNAs. Guide RNAs used within this study were gRNA3436 (GCCAAGCAGTCCCTGCAAAT), genomic position Chr. 1 207757577–207757596 (Horizon Genomics, CatNo HZGGA003436) and gRNA3437 (ACTCGTAAGTCCCATTTGCA), genomic position Chr. 1 207757590–207757609 (Horizon Genomics, CatNo HZGGA003437) both targeting the human CD46 locus on chromosome 1 coding for CD46 (Ref Seq: NM_153826). Both gRNAs are located adjacent to each other. For transfection in 60 mm dishes the DNA mix consisted of 3.6 μg Cas9 plasmid, 3.6 μg of the respective gRNA plasmid and 0.8 μg of the blasticidin-GFP plasmid.

Genomic DNA was isolated directly from cells in 96-well plates. 100 μl Direct PCR-Cell buffer (PEQLAB) supplemented with Proteinase K (400 μg/ml) was added to the cells and incubated for 3 hours at 56°C. Samples were heated for 45 minutes to 80°C for inactivation of Proteinase K. Thereof 1.5 μL were used as input for PCR to amplify genomic DNA prior to sequencing. Amplification of the CD46 locus for sequencing was done using flanking primers (fwd: ATATTCCCACCCATTCAAAAGAGCA; rev: CATCCAAGAGTTGTTTGGCTAGAAG). PCR was performed using GoTaq polymerase (Promega) according to the manufacturer’s instructions. In order to facilitate amplification from genomic DNA the PCR mix was supplemented with 1% DMSO. PCR products were purified using FavorPrep PCR purification Mini Kit (Favorgen). Purified PCR products having a minimum concentration of 5 ng/mL were sequenced at Eurofins Genomics. The forward PCR primer was also used for Sanger sequencing. Sequences were analyzed with SeqMan Pro software version 8.0.2 (Lasergene DNA Star).

### 5.3. Analysis of protein expression using immunofluorescence stainings

Analysis of expression of CD46 and CD13 was performed on live cells. For CD46 analysis 1 x 10^6^ cells per analysis were harvested, centrifuged and re-suspended in 1% BSA in PBS to block unspecific binding. APC-mouse anti-human CD46 antibody (BD Biosciences) and APC-mouse IgG2a κ Isotype control antibody were diluted 1:20 in blocking solution. DAPI (100 ng/mL) was used for exclusion of dead cells. For analysis of CD13 5 x 10^5^ cells were used per analysis, blocking was done in 5% FCS in PBS and mouse anti-Human CD13 antibody (Southern Biotechnologies) was diluted 1:100. Anti-mouse Alexa Fluor 488 antibody (Life Technologies) diluted 1:500 was used as secondary antibody. Analysis was performed on a Beckman Coulter FACS Gallios equipped with a Blue Solid State Diode (488 nm), a Red Solid State Diode (638 nm) and a Violet Solid State Diode (405 nm).

For detection of E-cadherin and ZO-1, cells were grown in IBIDI-slides and fixed in 3.6% paraformaldehyde. Blocking and permeabilization was done using 5% FCS, 0.2% Triton-X-100 in PBS. Staining was done overnight at 4°C using goat anti-human E-Cadherin antibody (R&D Systems) diluted 1:100 and rabbit anti-human ZO1 antibody (Thermo Scientific) diluted 1:100. Secondary antibodies used were anti-goat IgG Alexa Fluor 488 (Jackson Immuno Research) diluted 1:1000 and anti-rabbit IgG Alexa Fluor 594 (Jackson Immuno Research) diluted 1:500. Nuclei were counterstained with DAPI (100 ng/mL). Images were acquired by fluorescence microscopy on a Leica DMI-6000 microscope equipped with filter sets for DAPI, GFP and RFP.

### 5.4. Complement activation and co-factor activity assay

For increased HLA molecule surface expression control transfected or CD46 -/- RPTEC/TERT1 cells were stimulated for 48 hours with a cytokine cocktail consisting of IL-1β (5 ng/mL), TNF-α (1000 units/ml) and IFN-γ (300 units/ml) in ProxUp serum-free medium in 24 well culture plates. For C4b and C4d deposition assays, 90% confluent cells were incubated for one hour at 37°C with a pan-anti-HLA class I antibody (anti-HLA ABC, clone W6/32, Bio-Rad, Hercules, CA, USA), diluted 1:250 in an incubation mixture of 50%vol serum (as a source of complement and factor I) from a healthy volunteer with blood group AB and 50%vol ProxUp medium. For C3b and C3d deposition assays the same classical pathway activation setting was used but with a serum/ProxUp medium ratio of 25 to 75%vol. For flow cytometric analysis, cells were detached by incubating them with 10 mM EDTA in PBS for 10 min at 37°C, followed by a gentle treatment with a cell lifter. For each analysis, 5x10^4^ cells were stained either with R-PE-conjugated AffiniPure F(ab’)2 fragment donkey anti-mouse IgG (H+L) (final dilution 1:25; Jackson Immuno Research) or with biotinylated anti-human C4c, biotinylated anti-human C4d (final dilutions 1:20) or biotinylated anti-human C3d (final dilution 1:25; all from Quidel, San Diego, CA, USA) or with FITC-labeled rabbit anti-human C3c (final dilution 1:20; Dako, Glostrup, Denmark) on ice for 30 min. After washing with PBS, only those tubes containing biotinylated detection antibodies were further incubated with eBioscience Streptavidin PE (final dilution 1:100; Thermo Fisher Scientific, Waltham, MA, USA). Flowcytometric measurement was performed on a FACSCanto II equipped with FACSDiva Software Version 6.1.2 (BD Biosciences, San Jose, CA, USA) and FloJo Software Version 7.2.2 was used for further analysis. Results are presented as mean and standard deviation (C4b/d deposition assay: 4 experiments; C3b/d deposition assay: 6 experiments) of geometric mean of fluorescence intensity (geoMFI). Statistical differences and significance values were analyzed by two-sided t test considering Levene’s test of homogeneity of variances.

### 5.5. Gamma-glutamyl transferase activity

Gamma glutamyl transferase activity (GGT) was assayed as described previously [57]. In brief cells were grown to confluence in a 12-well format, 1 mM gamma-glutamyl para nitroanilide (GPNA) as a substrate for GGT was provided and the resulting yellow coloured cleavage product was measured at 405 nm. Obtained OD values were quantified using para-nitroanilide (pNA) as a standard and expressed as nmoles converted per minute.

## Supporting information

S1 Fig Analysis of CD46 knock-out: **a** Guide RNA 3436 transfected homozygous knock-out clone 1C3. **b** Guide RNA 3437 transfected heterozygous knock-out clone 1B10.
(TIF)
